# Physicochemical Quality and Flavor Characteristics of Sevenband Grouper (*Hyporthodus septemfasciatus*) Fillets: Effects of Boiling, Steaming, Oven Cooking, and Microwave Cooking

**DOI:** 10.3390/foods15152597

**Published:** 2026-07-24

**Authors:** Soomin Lee, Seyeon Park, Jimin Lim, Seungeun Lee, Jieun Oh

**Affiliations:** 1 Department of Nutritional Science and Food Management, Ewha Womans University, Seoul 03760, Republic of Korea; e_soomin@ewha.ac.kr (S.L.); seyeon315@ewha.ac.kr (S.P.); jiminlim@ewha.ac.kr (J.L.); yisseung_@naver.com (S.L.); 2 College of Science and Industry Convergence, Ewha Womans University, Seoul 03760, Republic of Korea

**Keywords:** sevenband grouper (*Hyporthodus septemfasciatus*), heat treatment, physicochemical property, volatile compound, flavor

## Abstract

Sevenband grouper (*Hyporthodus septemfasciatus*) is a premium aquaculture species with a high-protein, low-fat profile, yet cooking-related quality data remain limited. This study evaluated the effects of boiling, steaming, oven cooking, and microwave cooking on the physicochemical, flavor-related, and microstructural properties of grouper fillets. Weight loss differed significantly among methods (*p* < 0.001); oven cooking produced the greatest loss (32.42 ± 3.17%) and boiling the least (16.66 ± 3.89%). Shear index decreased after cooking; boiling produced the softest texture (75.57 ± 28.49 g·s), while steaming remained the firmest (192.46 ± 9.12 g·s; *p* < 0.001). pH and lightness (L*) increased after heating in most methods, but microwave-cooked samples showed pH similar to raw fish, whereas oven cooking produced the greatest yellowness (b* = 21.54 ± 0.44). E-nose analysis (PC1 = 53.79%, PC2 = 20.51%) separated moist-heat samples from oven-cooked samples. E-tongue analysis showed increased sourness after oven and microwave cooking and decreased saltiness after microwave cooking, while umami showed no clear change. Microstructural analysis showed the widest inter-fiber spacing in oven-cooked samples, matching greater moisture loss. These findings provide physicochemical, flavor-related, and microstructural data for sevenband grouper and support future processing and product development.

## 1. Introduction

Seafood is a major dietary source of protein, vitamins, minerals, and omega-3 fatty acids, and rising demand for these health benefits—reflected in a global per capita consumption of 20.7 kg in 2022, nearly double the level recorded approximately 50 years ago—has driven a corresponding expansion of seafood production [1,2,3]. To meet this growing demand, seafood production has expanded accordingly, with aquaculture emerging as a principal supply mechanism owing to its technological advantage of allowing controlled production conditions [4]. Reflecting this growing significance, aquaculture production accounted for 51% of global seafood output in 2022—surpassing capture fisheries for the first time [3]—and in Korea, aquaculture production has grown to approximately double that of wild-catch fisheries [5].

Within this context, interest in high-value aquaculture species has grown considerably. Among these, sevenband grouper (*Hyporthodus septemfasciatus*) is highly valued for its high-protein, low-fat nutritional profile, mild flavor, and favorable growth characteristics [6,7]. Domestic aquaculture of this species has been established for approximately two decades, and it has recently been designated as a strategic export species targeting key consumer markets, further expanding its potential for industrial utilization [8]. Nationwide production of sevenband grouper was reported at 269 tons in 2010 [7]. It commands a premium market price and is predominantly distributed and consumed live, being widely recognized as a popular ingredient for high-grade raw fish (*hoe*) preparation [9].

The palatability and quality of aquatic products are significantly influenced not only by nutrition but also by heat treatment reactions during the cooking process [10,11]. Protein denaturation and tissue changes directly affect the final flavor and texture of fish, which are closely related to consumer acceptance [10,11,12]. Understanding the physical and physicochemical properties of aquatic products under different heat treatment methods is therefore necessary for the advancement of aquatic product technology [13]. Previous studies have addressed such changes in other common fish species, including alterations in the fatty acid composition of black rockfish [14], compositional changes in tuna, mackerel, and horse mackerel [15,16], physicochemical and preference comparisons of grilled mackerel [17], texture and flavor changes in shrimp and scallops [18,19], proton dynamics and physicochemical attributes in Spanish mackerel under different cooking methods [20], and Maillard-reaction-derived volatile compounds in blue round scad enzymatic hydrolysate [21]. By contrast, previous research on grouper has focused primarily on aquaculture-related aspects, such as sex-reversal induction [22], ovarian development and maturation [23], maturation and ovulation induction [24], and immunological mechanisms [25], while its quality characteristics from a heat-treatment perspective remain unexplored.

Studies that systematically compare quality changes across actual consumption contexts—including raw preparation, moist-heat cooking, dry-heat cooking, and microwave cooking—are still lacking for this species. Oven cooking (dry heat), boiling and steaming (moist heat), and microwave cooking differ fundamentally in their heat transfer mechanisms and moisture environments, and are therefore expected to result in distinct effects on the physicochemical and flavor-related quality characteristics of sevenband grouper. Understanding quality changes under these cooking conditions is essential for reflecting common household and commercial cooking practices and for providing fundamental information for seafood processing design and ensuring product quality consistency.

We hypothesized that different heat treatment methods would induce distinct physicochemical, structural, and flavor-related changes in sevenband grouper, reflecting the inherent differences in cooking conditions among the methods applied. Therefore, this study systematically investigated the effects of boiling, steaming, oven cooking, and microwaving on the physicochemical, flavor, and microstructural properties of sevenband grouper, with the aim of providing foundational data to support processing standardization and product development.

## 2. Materials and Methods

### 2.1. Samples

Groupers (*Hyporthodus septemfasciatus*) weighing 2.0–2.5 kg and cultured for less than three years were purchased on 28–29 October 2024 from Buan Fisheries (Noryangjin Fish Market, Seoul, Republic of Korea). The fish were originally shipped from the Tongyeong Fisheries Cooperative (Tongyeong, Gyeongsangnam-do, Republic of Korea) and transported to the market in an ice-chilled insulated container. Fish were sacrificed at approximately 10:00 on the day of each experiment. Based on a previous study indicating that storage within 6 h does not exceed provisional standards for aquatic products [26], all subsequent sample preparation and analyses were completed within 5 h (i.e., by approximately 15:00). Upon arrival at the laboratory, samples were stored under refrigeration at 5 °C in a household refrigerator (T9000, RF85M9002W1, Samsung Electronics, Suwon, Republic of Korea) prior to analysis, in accordance with the Korea Food Code standard specifying that raw fish intended for direct consumption be maintained at or below 5 °C [27].

A total of four grouper individuals were obtained and used across the study. Because the tissue quantity required differed among analyses, three individuals were allocated to each measurement (e.g., weight loss, texture, pH, color, e-nose, e-tongue, proximate and fatty acid composition, and SEM), drawn from this shared pool of four; the specific set of three individuals varied somewhat among measurement types. For each individual, several tissue pieces were prepared per cooking treatment, and the mean value across these pieces was taken as that individual’s representative value. The triplicate (n = 3) values reported in this study therefore correspond to three biological replicate individuals per measurement, with standard deviations reflecting inter-individual variability.

### 2.2. Sample Preparation

Four heat treatment methods were selected: boiling, steaming, oven cooking, and microwave cooking. Samples were prepared from the loin portion of the fish and standardized to dimensions of 5 cm (length) × 3 cm (width) × 2 cm (height). The endpoint of heat treatment was defined as a cooked state corresponding to a core temperature of 63 °C, in accordance with guidelines for fish products [28]. Cooking times for each method were determined through preliminary experiments based on internal temperature measurements, and all cooking methods were confirmed to reach the target core temperature of 63 °C. Based on the general principle that short-duration heating better preserves the inherent flavor of fish, whereas prolonged heating leads to flavor deterioration [29], the maximum cooking time in this study was set at 15 min.

Samples were immersed in 200 mL of boiling water and heated for 15 min using an induction cooktop (DIH-180B, Daeryung, Gwangju, Republic of Korea) operated at 1800 W. Samples for steaming were placed on a cheesecloth-lined perforated steam basket insert and heated for 15 min over boiling water on an induction cooktop operated at 1800 W, allowing direct steam exposure to the samples. Oven cooking was performed in a preheated convection oven (Convotherm MAXX 6.10, Convotherm, Eglfing, Germany) at 195 °C for 10 min. Microwave cooking was carried out in a microwave-safe ceramic container without a cover using a microwave oven (MS23K3513AW, Samsung Electronics, Suwon, Republic of Korea) at 1100 W for 2 min.

### 2.3. Weight Loss (%)

Weight loss (%) was calculated as the percentage decrease in sample weight after heat treatment. Each sample was weighed immediately before and after heat treatment, and weight loss was determined using the following equation:

$$Weight\;loss\;\left(\%\right)=\frac{\left(Weight\;before\;heat\;treatment-Weight\;after\;heat\;treatment\right)}{Weight\;before\;heat\;treatment}\times 100$$

### 2.4. Color

Color measurements were conducted using a spectrophotometer (CM-700d, Konica Minolta, Tokyo, Japan). Hunter color values L* (lightness), a* (redness/greenness), and b* (yellowness/blueness) were recorded. The instrument was calibrated using a standard white plate prior to measurement. Color measurements were performed after allowing cooked samples to cool at room temperature for 5 min, in order to minimize surface moisture condensation on the sensor and to standardize measurement conditions across cooking methods with different surface temperatures immediately after cooking. For each individual, one measurement point was taken per tissue piece; three tissue pieces were measured and averaged to obtain that individual’s representative value, following the replicate structure described in Section 2.1.

### 2.5. Texture

Texture analysis was performed using a texture analyzer (TA-XT2i, Stable Micro Systems, Godalming, UK) equipped with a Mini Kramer shear cell probe. After heat treatment, samples were allowed to cool at room temperature for 5 min, using the same cooling protocol applied for color and pH measurements, and were then cut into 1.5 cm × 1.5 cm × 2.5 cm pieces and tested at a pre-test speed of 2 mm/s, test speed of 2 mm/s, and post-test speed of 2 mm/s. The area under the force–time curve (g·s) was used as an instrumental shear index (hereafter referred to as the shear index), reflecting the relative resistance of the sample to compression and shear.

### 2.6. pH

The pH of samples was measured in accordance with the Food Code [27]. Briefly, 15 g of sample was homogenized with nine volumes of distilled water for 2 min using a homogenizer (T18 Basic, Ultra-Turrax^®^, IKA^®^, Staufen, Germany). The homogenate was filtered through qualitative filter paper (No. 2, Advantec, Tokyo, Japan), and the filtrate was measured three times using a pH meter (Orion Star A211, Thermo Scientific, Singapore).

### 2.7. E-Nose Analysis

Volatile odor profiles were analyzed using an electronic nose system (HERACLES Neo, Alpha MOS, Toulouse, France). Cooked samples were allowed to cool at room temperature for 5 min prior to sub-sampling, following the same protocol used for the other measurements. Three grams of each sample were then placed in a 20 mL headspace vial and equilibrated at 40 °C for 20 min with agitation at 500 rpm. Volatile compounds were captured using an automatic sampler. Analysis conditions were as follows: acquisition time, 93 s; trap absorption temperature, 40 °C; trap desorption temperature, 250 °C; hydrogen carrier gas flow rate, 1 mL/min. Separation was performed using MXT-5 and MXT-1701 columns connected in parallel, and detection was carried out using a flame ionization detector. Data were processed using AlphaSoft software (ver. 14.2, Alpha MOS), and retention indices were calculated based on the Kovats index library. Volatile compounds were tentatively identified by comparing retention indices with those in the AroChemBase database (Alpha MOS). The relative abundance of volatile compounds was expressed as peak area obtained from the chromatographic signal. As the applied e-nose system provides rapid, pattern-based volatile profiling rather than compound-specific confirmation, the resulting volatile profiles in this study should be interpreted as semi-quantitative screening data rather than confirmed chemical identities.

### 2.8. E-Tongue Analysis

For e-tongue analysis, 15 g of sample was homogenized with nine volumes of purified water for 2 min. The homogenized solution was filtered using filter paper (No. 2, 110 mm; Advantec Toyo Kaisha, Tokyo, Japan) under a clean bench to remove solid particles and then diluted 10000-fold. An aliquot (80 mL) of the diluted solution was analyzed using an electronic tongue system (Astree 2, Alpha MOS, Toulouse, France) equipped with seven sensors (AHS, CTS, NMS, PKS, ANS, CPS, and SCS). Sensor responses were processed using Alpha MOS software (Alpha MOS, Toulouse, France), which converts sensor signal patterns into taste scores ranging from 0 to 12. Similarly, as the e-tongue system provides pattern-based taste profiling rather than direct quantification of taste-active substances, the resulting taste profiles in this study should be interpreted as semi-quantitative screening data rather than as absolute concentrations of specific compounds. Following the sensor configuration adopted in a previous electronic tongue application for fish-based food products [30], NMS, CTS, and AHS were interpreted as sensors corresponding to umami, saltiness, and sourness, respectively, whereas PKS, ANS, CPS, and SCS were used as standard (reference) sensors.

### 2.9. Microstructure

Microstructural observation was performed using a scanning electron microscope (SEM). Samples were freeze-dried at −80 °C for 96 h using a freeze dryer (FD8518, ilShinBioBase, Dongducheon, Republic of Korea). The dried samples were mounted on stubs with carbon tape, sputter-coated with gold (SCM-200, Polaris, Suwon, Republic of Korea) at 10 mA for 90 s under vacuum, and observed using an SEM (SU-70, Hitachi, Tokyo, Japan) at an accelerating voltage of 10 kV and 100× magnification.

### 2.10. Statistical Analysis

Data were expressed as mean ± standard deviation (SD). Statistical analysis was performed using SPSS 31.0 Statistics software (IBM, Armonk, NY, USA). Differences among samples were evaluated using one-way analysis of variance (ANOVA), followed by Tukey’s honestly significant difference (HSD) test at a significance level of *p* < 0.05. Principal component analysis (PCA) was conducted to visualize differences among samples based on volatile compound profiles. All measurements were performed in triplicate. For each measurement, the experimental unit for statistical analysis was the individual fish; three biologically independent individuals were analyzed per measurement, with each individual’s value representing the mean of multiple tissue sub-samples.

## 3. Results and Discussion

### 3.1. Weight Loss

After heat treatment, the weight decreased in all samples (Table 1). Weight loss (%) varied significantly depending on the heat treatment method (F = 19.732, *p* < 0.001). The highest weight loss was observed during oven cooking (32.42 ± 3.17%), followed by microwave cooking (18.79 ± 0.92%), steaming (18.53 ± 2.49%), and boiling (16.66 ± 3.89%).

These differences in weight loss are believed to be due to moisture loss during the heat treatment process [31]. Moisture loss occurs through evaporation and dripping, which are common in fish [32]. Approximately 80% of fish muscle consists of water, and heating induces changes in myofibrillar proteins and membrane structures, thereby reducing water-holding capacity and leading to moisture loss [33].

Grouper baked in dry-heat environments (oven) had greater weight loss than in wet-heat environments (steaming and boiling). This is likely attributable to the presence of external moisture in wet-heat environments, which suppresses surface evaporation and reduces overall moisture loss during cooking [34]. Despite higher fat retention observed in oven-cooked samples (Table A1), the greater weight loss is primarily attributed to extensive moisture evaporation under dry heating conditions rather than lipid loss. This trend is consistent with a previous study [35], which showed that dry-heat cooking methods result in significant moisture loss.

Overall, oven-cooked samples exhibited greater weight loss than those cooked by wet-heat methods (steaming and boiling), while microwave-treated samples showed intermediate behavior, reflecting moisture-mediated heating characteristics. This highlights the importance of selecting appropriate heat treatment conditions when product yield is a key processing objective.

### 3.2. Texture

The shear index of all heat-treated samples decreased compared with that of raw fish (Table 1). Among the heat treatment methods, boiling resulted in the lowest shear index value (75.57 ± 28.49 g·s), whereas steaming showed the highest value (192.46 ± 9.12 g·s), indicating that texture varied depending on the cooking method (F = 35.675, *p* < 0.001).

The mechanical strength of fish muscle is primarily governed by the structural integrity of muscle fibers and the connective tissue matrix that maintains muscle shape [36]. Heat treatment weakens muscle structure mainly through the thermal conversion of collagen, a key component of connective tissue, into gelatin, resulting in softening and reduced resistance to external forces [36,37]. Accordingly, the overall decrease in shear index observed after heat treatment in this study is likely attributable to changes in muscle structure, myofibrillar proteins, and connective tissue induced by heating.

The lowest shear index value observed after boiling is likely associated with extensive collagen gelatinization under high-temperature, water-mediated conditions, which accelerates connective tissue breakdown and muscle softening [38]. This finding agrees with previous observations that heat treatment promotes loosening of muscle fibers and tissue softening through structural collapse and collagen dissolution [39].

In contrast, oven-cooked and microwave-treated samples exhibited higher shear index values than boiled samples, with oven-cooked samples showing higher values than microwave-cooked samples. Dry-heat cooking methods, which do not use water as a heat transfer medium, can promote moisture loss and induce condensation and shrinkage of muscle fiber proteins during heating. This process may increase tissue rigidity despite the overall softening effect associated with collagen melting.

Interestingly, steaming resulted in the highest shear index among all heat treatment methods. Although steaming is classified as a wet-heat method, its relatively low heat transfer efficiency compared with boiling may limit the extent to which muscle fibers and connective tissue reach temperatures sufficient for collagen denaturation and structural collapse. Given the relatively short heat treatment time and the limited penetration of effective denaturation temperatures, the connective tissue structure may have been partially preserved, contributing to the relatively high residual shear index observed in steamed samples [37,38].

**Figure 1:**
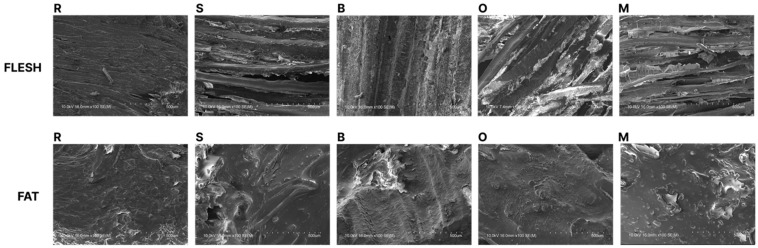
Microstructure of freeze-dried sevenband grouper subjected to different cooking methods. (R, raw; S, steaming; B, boiling; O, oven; M, microwave; FLESH, myotomal white muscle; FAT, lipid-rich region; Scale bar = 500 µm).

Taken together, these results demonstrate that texture changes in grouper are strongly influenced by heat transfer mechanisms and moisture dynamics associated with different cooking methods. These texture changes did not, however, consistently parallel the microstructural findings (Figure 1): boiled samples retained comparatively narrow inter-fiber spacing despite their pronounced textural softening, whereas the widest spacing occurred in oven-cooked samples as a result of moisture loss. The low shear index of boiled samples is thus considered to reflect softening at the connective-tissue level, while the limited inter-fiber spacing observed by SEM is considered to reflect a separate aspect of heat-induced change, namely the spatial separation of muscle fibers.

### 3.3. Color

The color values of grouper before and after heat treatment are presented in Table 1. Lightness (L*), redness (a*), and yellowness (b*) ranged from 39.63 ± 2.33 to 79.31 ± 2.04, −4.29 ± 0.23 to −1.64 ± 0.68, and −1.72 ± 0.99 to 21.54 ± 0.44, respectively. Microwave-cooked grouper exhibited the highest L* value, followed by boiled, steamed, oven-cooked, and raw samples. The highest a* value was observed in oven-cooked samples, whereas boiled and microwave-cooked samples showed the lowest a* values, with raw samples showing an intermediate value. Oven-cooked grouper showed the highest b* value, while the lowest b* value was recorded in raw fish. Color values varied significantly depending on the heat treatment method (L*: F = 48.470, *p* < 0.001; a*: F = 19.870, *p* < 0.001; b*: F = 97.246, *p* < 0.001).

These color changes may be attributed to multiple heat-induced mechanisms. When fish muscle is cooked, the flesh becomes opaque and lighter in appearance, which is presumed to be mainly due to protein coagulation leading to light scattering [40]. More specifically, color changes are presumed to be associated with the oxidation of heme proteins, hemoglobin and myoglobin [41], which is closely related to myoglobin content and solubility in muscle foods [42,43]. In fish, myoglobin exists predominantly as bright-red oxymyoglobin, but heating oxidizes the heme moiety [44], forming pale-colored derivatives that shift the flesh toward white and increase lightness [10]. This mechanism likely accounts for the decrease in a* (redness) and increase in L* (lightness) observed after cooking.

Notably, oven-cooked samples exhibited relatively low L* values and the highest b* values among the heat-treated groups, indicating pronounced yellowness and browning, consistent with similarly pronounced browning and grayish discoloration reported under low-moisture heating conditions [45,46]. By contrast, oven-cooked samples retained the highest a* value among the treated groups, suggesting that browning-related pigment formation, rather than myoglobin-driven loss of redness, may have been the dominant color-determining mechanism under this dry-heat condition. This pattern suggests that Maillard-type browning reactions, which darken fish color through brown pigment formation, may have occurred alongside reduced light reflectance resulting from moisture loss during processing [47,48]. In line with the weight loss results (Section 3.1), the greater moisture reduction observed during oven cooking may have intensified these surface browning reactions and altered light reflectance, likely contributing to the distinctive color characteristics of oven-treated grouper. This is also in line with previous findings that Maillard reactions proceed more actively under dry-heat than moist-heat cooking [48], and that lower moisture content is associated with enhanced Maillard reaction rates [45].

### 3.4. pH

The pH of grouper before and after heat treatment is shown in Table 1. Among the heat-treated samples, steaming showed the highest pH (7.18 ± 0.04), followed by boiling (7.00 ± 0.02), oven cooking (6.96 ± 0.02), and microwave cooking (6.70 ± 0.09), while raw grouper exhibited the lowest pH (6.70 ± 0.06). Overall, heat treatment tended to increase the pH of the samples, with the exception of microwave cooking. These results are similar to those reported for other heat-treated fish species [10,49] and for sea snail meat [50].

This increase in pH can be explained by heat-induced protein denaturation, which may expose basic groups and suppress acidic groups as protein structure changes during heating [51]. Heat treatment may also alter amino-acid side chains and promote reactions involving them [52], and other heat-induced reactions may consume or modify functional groups, thereby influencing pH [53,54]. Taken together, these findings indicate that pH shifts in cooked grouper reflect heat-driven changes in muscle protein chemistry, with the magnitude of change varying by heat treatment method, remaining minimal under the markedly shorter heating time of microwave cooking relative to the other methods.

### 3.5. E-Nose Analysis

**Figure 2:**
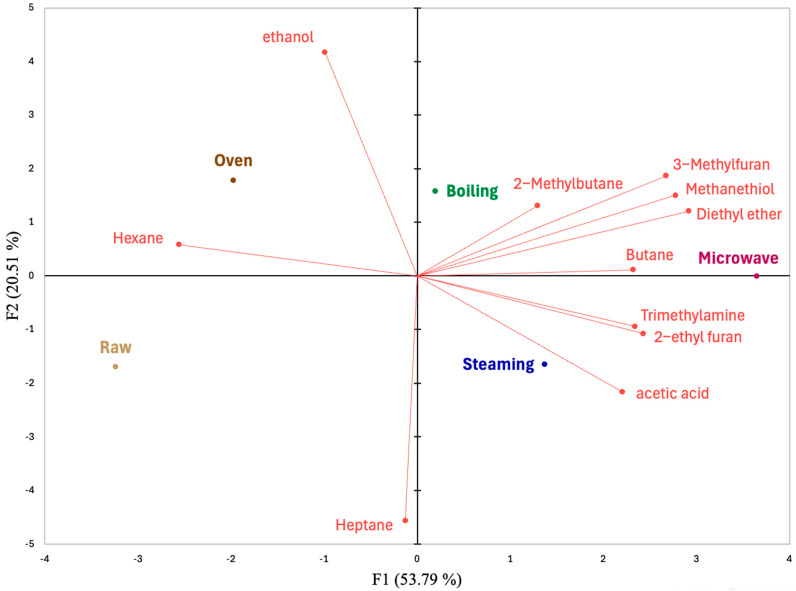
Principal component analysis (PCA) of e-nose profiles of sevenband grouper (*Hyporthodus septemfasciatus)* by cooking method. (PC1 and PC2 = first and second principal components).

In this study, 11 major volatile compounds with the highest discrimination power (DP) values among the detected volatile compounds were selected, and the results are summarized in Table 2. The DP value is an index used to represent the magnitude of differences among samples based on volatile profiles [55]. These compounds were classified into one alcohol, one acid, one amine, one ether, two furans, four hydrocarbons, and one sulfur compound. Principal component analysis (PCA) was conducted using these 11 indicator compounds to compare aroma patterns according to heat treatment methods (Figure 2). PC1 and PC2 explained 53.79% and 20.51% of the total variance, respectively, with a cumulative explanatory power of 74.30%, indicating clear differentiation among samples by heat treatment method.

Boiled and steamed samples were located in the positive direction of PC1 and were associated with volatile compounds such as methanethiol (F = 4.834, *p* < 0.05), 2-ethylfuran (F = 6.236, *p* < 0.01), and acetic acid (F = 4.781, *p* < 0.05). Among these, 2-ethylfuran has been reported as a representative indicator of lipid oxidation during thermal processing of fish, with its formation increasing as heating intensity increases [56], consistent with reports that furan generation increases more broadly in heated foods [57,58]. The association of sulfur- and acid-related compounds with these wet-heat treatments may reflect the promotion of lipid oxidation and amino-acid-related degradation pathways during heating.

Trimethylamine (TMA) (F = 4.304, *p* < 0.05) was positioned in the negative PC1 and positive PC2 quadrants in the PCA biplot. This distribution matches the thermal decomposition and release of trimethylamine-N-oxide (TMAO) during fish heat processing, a characteristic reaction pathway in marine fish species [59]. Although elevated TMA levels are often associated with undesirable odors, their presence contributes to the overall flavor profile formed during heat treatment [60].

Oven-cooked samples were clearly separated from other treatments and were positioned in the positive PC1 and negative PC2 quadrants, showing a strong association with ethanol (F = 0.512, *p* > 0.05), although this difference did not reach statistical significance. This distribution reflects the influence of prolonged exposure to dry heat, which promotes dehydration and lipid oxidation at the surface, leading to the generation and retention of secondary volatile compounds such as alcohols [61], consistent with related observations in other dry-heat-cooked foods [62]. This pattern may also relate to a previously reported decrease in acetic acid concentration with increasing oven temperature, attributed to a relative increase in Maillard reaction products [63]; consistent with this, acetic acid content in the oven-cooked samples of the present study (47.86 ± 44.68) was lower than in the raw samples (99.58 ± 16.63; Table 2). Notably, oven-cooked and microwave-treated samples were distributed in distinct regions of the PCA space, indicating that differences in heat transfer mechanisms and heating dynamics resulted in divergent aroma-forming pathways.

### 3.6. E-Tongue Analysis

**Figure 3:**
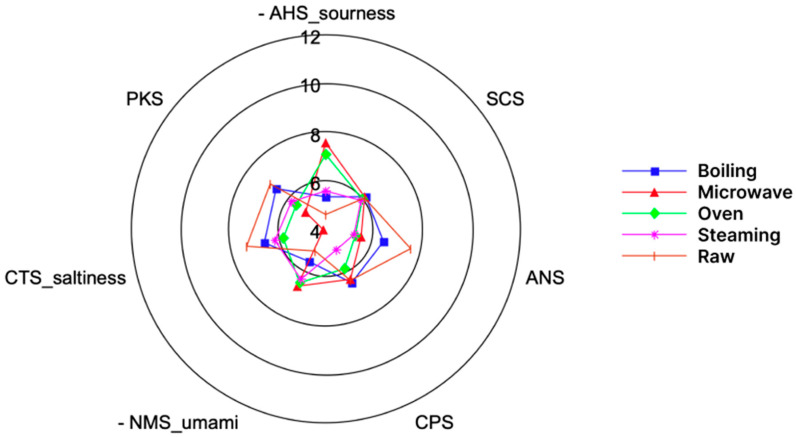
E-tongue taste intensity profiles (0–12 scale) of sevenband grouper (*Hyporthodus septemfasciatus)* subjected to different cooking methods. The electronic tongue system was equipped with seven sensors.

**Figure 4:**
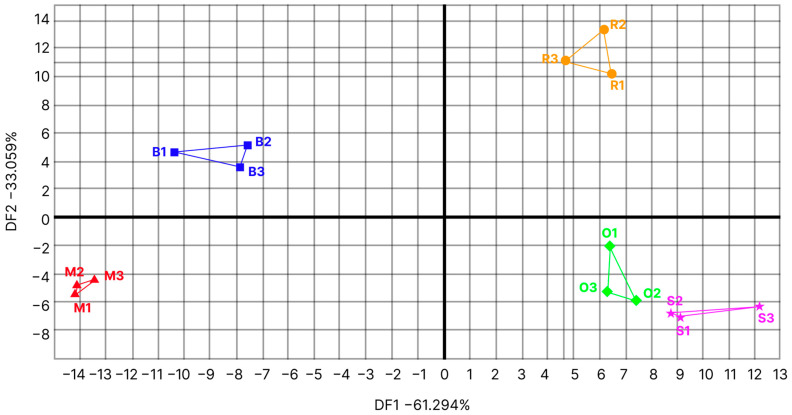
Discriminant function analysis (DFA) plot of electronic tongue sensor responses for sevenband grouper (*Hyporthodus septemfasciatus*) fillets subjected to different cooking methods.

Figure 3 presents the radar chart based on the converted scores of the AHS (sourness), CTS (saltiness), and NMS (umami) sensors, alongside the discriminant function analysis (DFA) plot (Figure 4) and the underlying sensor response values (Table 3). Sourness (AHS) scored 4.60 in the raw group and rose progressively to 5.30, 5.60, 7.00, and 7.50 in the boiling, steaming, oven, and microwave groups. Saltiness (CTS), by contrast, was highest in the raw group (7.30) and declined to 6.60, 6.30, 5.80, and 4.10 under the same four treatments. Umami (NMS) followed a pattern similar to sourness, increasing from 5.00 in the raw group to 5.50, 5.00, 6.50, and 6.70 after boiling, steaming, oven cooking, and microwaving, respectively.

Not all of these numerical shifts, however, translate into a perceivable difference in taste. A reactivity difference of at least 2.0 has been reported as the threshold required for a human taster to detect a change in intensity [64]. Applying this threshold, only the oven- and microwave-cooked groups showed a sourness increase large enough to be perceivable (differences of 2.40 and 2.90 from the raw group), while the boiling and steaming groups (0.70 and 1.00) fell well short. For saltiness, only microwave cooking produced a perceivable reduction (a difference of 3.20), whereas the other three methods (0.70–1.50) would not register as different to a human taster. None of the umami shifts reached the 2.0 threshold, indicating that this attribute would be perceived as essentially unchanged regardless of cooking method.

The perceivable rise in sourness after oven and microwave cooking is consistent with reports that heat treatment can degrade succinic acid in aquatic muscle tissue while generating new organic acids such as lactic and malic acid [65], a shift that would be expected to sharpen sour notes. The perceivable drop in saltiness under microwave cooking, meanwhile, aligns with earlier findings of sodium and potassium loss in cooked fish fillets [66]. As the compounds responsible for these sensory responses were not directly measured in the present study, these explanations should be regarded as plausible rather than confirmed.

### 3.7. Microstructure

The microstructure of grouper samples before and after heat treatment was observed using scanning electron microscopy (SEM), as shown in Figure 4. Prior to heat treatment, the muscle surface appeared smooth and compact, with no apparent fiber separation or cracks. After heat treatment, structural disruption became evident, characterized by fiber separation and cracking, similar to previously reported observations in heat-treated fish muscle [20].

The degree of microstructural damage increased in the following order: boiling, steaming, microwave cooking, and oven cooking. Overall, oven cooking resulted in the most pronounced fiber separation, followed by microwave cooking, whereas moist-heat treatments (boiling and steaming) showed comparatively less structural disruption, with the oven-cooked samples exhibiting the widest inter-fiber spacing. These structural changes are consistent with the extent of moisture loss observed under each cooking condition.

Compared with the findings of Sun et al. [20], similar microstructural trends were observed in oven-treated samples; however, differences were noted in the extent of structural disruption. These discrepancies may be attributed to differences in sample preparation and experimental design. In the previous study, heat treatment groups were randomly selected after sample cutting, whereas in the present study, all treatments were applied to standardized loin cuts from the same anatomical region. Variations in local fat distribution within randomly selected samples in earlier studies may have contributed to the observed differences in microstructural response.

## 4. Conclusions

This study systematically evaluated the effects of boiling, steaming, oven cooking, and microwave cooking on the physicochemical, flavor, and microstructural properties of sevenband grouper (*Hyporthodus septemfasciatus*) fillets. Oven cooking resulted in the greatest weight loss and pronounced yellowing, while boiling produced the softest texture among all cooking methods. Heat treatment increased pH and lightness (L*), with the exception of pH in microwave-cooked samples, across all methods. E-nose analysis revealed distinct volatile profiles among cooking methods, with moist-heat treatments associated with sulfur- and acid-related compounds and oven cooking associated with ethanol. E-tongue analysis indicated a perceivable increase in sourness under oven and microwave cooking and a perceivable decrease in saltiness under microwave cooking, whereas umami remained largely unchanged. Microstructural observations were consistent with the physicochemical findings, showing the greatest fiber disruption in oven-cooked samples.

Several limitations should be acknowledged. This study was restricted to a single species, limiting generalizability across fish species with different muscle compositions and thermal responses. The four cooking methods also differed in heating time, power input, water contact, and heating environment, which makes it difficult to attribute the observed quality differences solely to the type of cooking method. This study also focused on conventional heat treatment methods, which may not fully represent modern industrial processing techniques such as smoking, freezing–thawing cycles, or high-pressure processing. In addition, different subsets of individuals were allocated across the various analyses, which prevented direct within-individual correlations between measurement types (e.g., texture and volatile profile) from being established. Continuous core-temperature-versus-time profiles and within-sample spatial temperature gradients were also not recorded, with only the attainment of the 63 °C endpoint verified for each sample. Proximate composition and fatty acid contents (Appendix A Table A1) were measured once per cooking method by an accredited external laboratory, without biological replication, precluding statistical comparison across treatments. Future studies should expand comparative analyses across multiple fish species and advanced processing technologies, incorporating fully paired experimental designs and continuous thermal monitoring, to further enhance the applicability of the preliminary quality data established in this study.

## Figures and Tables

**Table 1:** Weight loss, shear index, pH, and color values of sevenband grouper (*Hyporthodus septemfasciatus)* by different cooking methods.

		Raw	Cooking Method				F/*P* ^(4)^
			Steaming	Boiling	Oven	Microwave	
Weight loss (%)		- ^(2)^	18.53 ± 2.49 ^a (1) (3)^	16.66 ± 3.89 ^a^	32.42 ± 3.17 ^b^	18.79 ± 0.92 ^a^	19.732 ***
Shear index (g.s)		350.87 ± 0.10	192.46 ± 9.12 ^c^	75.57 ± 28.49 ^a^	175.87 ± 2.09 ^c^	130.48 ± 4.80 ^b^	35.675 ***
pH		6.70 ± 0.06	7.18 ± 0.04 ^c^	7.00 ± 0.02 ^b^	6.96 ± 0.02 ^b^	6.70 ± 0.09 ^a^	46.472 ***
Color ^(5)^	L*	39.63 ± 2.33	70.90 ± 1.78 ^b^	77.93 ± 0.86 ^c^	64.31 ± 1.95 ^a^	79.31 ± 2.04 ^c^	48.470 ***
	a*	−2.02 ± 0.46	−3.25 ± 0.47 ^b^	−4.29 ± 0.23 ^a^	−1.64 ± 0.68 ^c^	−4.09 ± 0.37 ^a^	19.870 ***
	b*	−1.72 ± 0.99	6.88 ± 1.67 ^b^	7.99 ± 0.84 ^c^	21.54 ± 0.44 ^d^	7.27 ± 1.59 ^b^	97.246 ***

^(1)^ Mean ± standard deviation; ^(2)^ “–” indicates “not applicable” because the raw sample was not heat-treated; ^(3)^ Different letters (a–d) within the same row indicate significant differences (*p* < 0.05) by Tukey’s honestly significant difference (HSD) test; ^(4)^ F values from one-way ANOVA. *** *p* < 0.001; ^(5)^ Color parameters: L* = lightness, a* = redness, b* = yellowness.

**Table 2:** Volatile compounds of sevenband grouper (*Hyporthodus septemfasciatus)* by different cooking methods.

Compounds	Sensory Descriptors ^(1)^	Raw	Cooking Method				F/*P* ^(6)^
			Steaming	Boiling	Oven	Microwave	
Alcohols:							
ethanol	Alcoholic; Ethanol; Pungent; Strong; Sweet; Weak	35.25 ± 61.06 ^(2) (3)^	63.60 ± 7.69	31.47 ± 54.51	51.04 ± 44.69	16.82 ± 29.14	0.512
Acids:							
acetic acid	Acetic; Acidic; Odorless; Pungent; Sharp; Sour; Vinegar	99.58 ± 16.63 ^ab (5)^	188.15 ± 51.18 ^b^	208.94 ± 156.14 ^ab^	47.86 ± 44.68 ^a^	311.09 ± 58.05 ^ab^	4.781 *
Amines:							
trimethylamine	Amine; Ammoniacal; Fishy; Fruity; Oily; Pungent; Rancid; Sweaty	661.00 ± 117.03 ^ab^	593.56 ± 171.84 ^ab^	1092.82 ± 264.09 ^a^	636.22 ± 47.79 ^a^	724.53 ± 164.04 ^b^	4.304 *
Ethers:							
diethyl ether	Etheral	176.11 ± 19.64 ^a^	304.13 ± 59.27 ^ab^	366.30 ± 82.33 ^ab^	232.18 ± 36.06 ^ab^	285.53 ± 37.90 ^b^	5.838 *
Furans:							
2-ethyl furan	Acidic; Burnt; Burnt (sweet); Chemical; Earthy; Malty; Pungent; Rubber; Sweet	ND ^a (4)^	140.45 ± 36.8 ^b^	155.55 ± 142.67 ^ab^	20.1 ± 34.81 ^a^	247.13 ± 48.03 ^ab^	6.236 **
3-Methylfuran		77.14 ± 67.64	332.21 ± 171.53	575.21 ± 355.62	294.75 ± 111.58	276.95 ± 112.17	2.555
Hydrocarbons:							
butane	Faint	677.09 ± 93.82 ^a^	690.16 ± 200.15 ^ab^	1289.58 ± 414.48 ^ab^	736.15 ± 24.24 ^ab^	724.69 ± 167.07 ^b^	4.122 *
heptane	Alkane; Fruity; Gasoline; Sweet	96.9 ± 39.36	39.71 ± 68.79	72.48 ± 63.02	35.86 ± 31.23	73.58 ± 86.58	0.526
hexane	Alkane; Etheral; Gasoline; Kerosene	35.45 ± 61.4	ND	ND	43.63 ± 37.78	ND	1.378
2-methylbutane	Gasoline; Pleasant	180.51 ± 34.19 ^a^	258.28 ± 38.46 ^b^	278.39 ± 48.96 ^ab^	329.5 ± 27.64 ^b^	344.37 ± 37.85 ^ab^	8.779 **
Sulfur compounds:							
methanethiol	Cabbage; Cabbage (cooked); Cheese; Egg (rotten); Fishy; Garlic; Gasoline; Meaty; Pungent; Rotten; Sulfurous; Unpleasant	1770.34 ± 514.96 ^a^	5839.50 ± 1989.99 ^ab^	8162.12 ± 3167.80 ^ab^	3319.99 ± 1115.21 ^ab^	4241.85 ± 1759.36 ^b^	4.834 *

^(1)^ Sensory descriptors were obtained from the instrument library (Alpha MOS) and AroChembase; ^(2)^ Mean peak area ± standard deviation; ^(3)^ Peak areas were obtained from the chromatographic signal using the electronic nose system software, and relative abundance was expressed as semi-quantitative data without the use of an internal standard; ^(4)^ ND = Not detected; ^(5)^ Different letters (a, b) within the same row indicate significant differences (*p* < 0.05) by Tukey’s honestly significant difference (HSD) test; ^(6)^ F values from one-way ANOVA. * *p* < 0.05, ** *p* < 0.01.

**Table 3:** Electronic tongue sensor response values of sevenband grouper (*Hyporthodus septemfasciatus*) fillets by different cooking methods (AHS, sourness; CTS, saltiness; NMS, umami; PKS, ANS, CPS and SCS, reference sensors).

	Raw	Cooking Method				F/*P* ^(3)^
		Steaming	Boiling	Oven	Microwave	
AHS (Sourness)	909.20 ± 50.29 ^(1)^	869.70 ± 35.93	879.02 ± 37.66	833.87 ± 24.31	815.61 ± 19.14	3.340
PKS	2694.66 ± 0.10	2543.84 ± 9.12	2619.22 ± 28.49	2544.92 ± 2.09	2493.36 ± 4.80	3.071
CTS (Saltiness)	1879.22 ± 4.69 ^b (2)^	1806.47 ± 18.60 ^b^	1830.80 ± 11.55 ^b^	1796.31 ± 15.73 ^b^	1658.55 ± 94.36 ^a^	10.538 ***
NMS (Umami)	1387.38 ± 182.06	1124.14 ± 126.23	1338.90 ± 152.22	1122.83 ± 100.41	1111.62 ± 120.06	2.810
CPS	526.24 ± 264.26	704.42 ± 146.50	648.27 ± 120.59	454.16 ± 563.15	663.72 ± 114.27	0.378
ANS	2559.89 ± 470.24 ^c^	2354.90 ± 48.62 ^a^	2503.06 ± 18.78 ^bc^	2350.97 ± 44.64 ^a^	2412.09 ± 27.98 ^ab^	16.616 ***
SCS	2054.56 ± 325.48	2135.25 ± 345.41	2100.01 ± 313.02	2197.00 ± 315.91	2295.13 ± 319.67	0.249

^(1)^ Mean ± standard deviation; ^(2)^ Different letters (a–c) within the same row indicate significant differences (*p* < 0.05) by Tukey’s honestly significant difference (HSD) test; ^(3)^ F values from one-way ANOVA. *** *p* < 0.001.

## Data Availability

The original contributions presented in this study are included in the article. Further inquiries can be directed to the corresponding author.

## Appendix A. Composition Metrics for Sevenband Grouper Processing

**Table A1:** Proximate and fatty acid compositions of sevenband grouper (*Hyporthodus septemfasciatus*) across cooking methods.

		Raw	Steaming	Boiling	Oven	Microwave
Calories (kcal/100 g)		153.12	137.56	120.84	176.87	150.47
Carbohydrate (g/100 g)		0.19	0.36	0.27	0.30	0.59
Lipid (g/100 g)		7.60	3.88	3.16	5.59	4.95
Protein (g/100 g)		20.99	25.30	22.83	31.34	25.89
Ash (%)		1.19	1.25	0.83	1.85	1.49
Moisture (%)		70.03	69.21	72.91	60.92	67.08
Abbreviation	Common Name	Raw (g/100 g)	Steaming (g/100 g)	Boiling (g/100 g)	Oven (g/100 g)	Microwave (g/100 g)
C12:0	Lauric acid	0.000	0.000	0.000	0.000	0.002
C14:0	Myristic acid	0.207	0.104	0.073	0.170	0.155
C15:0	Pentadecanoic acid	0.023	0.012	0.008	0.019	0.018
C16:0	Palmitic acid	1.224	0.680	0.494	1.076	0.967
C17:0	Margaric acid	0.026	0.014	0.010	0.023	0.021
C18:0	Stearic acid	0.285	0.169	0.127	0.273	0.237
C20:0	Arachidic acid	0.016	0.008	0.006	0.014	0.013
C21:0	Heneicosanoic acid	0.003	0.000	0.000	0.003	0.002
C22:0	Behenic acid	0.005	0.003	0.002	0.005	0.005
C23:0	Tricosanoic acid	0.002	0.001	0.000	0.002	0.002
C24:0	Lignoceric acid	0.005	0.003	0.002	0.005	0.004
Saturates		30.55	30.31	30.61	30.00	30.28
C14:1	Myristoleic acid	0.002	0.000	0.000	0.002	0.002
C16:1	Palmitoleic acid	0.367	0.187	0.132	0.308	0.276
C18:1, trans	Oleic acid	0.030	0.016	0.012	0.028	0.021
C18:1, cis		1.791	0.956	0.678	1.588	1.411
C20:1	Gadoleic acid	0.130	0.069	0.048	0.118	0.102
C22:1n-9	Erucic acid	0.022	0.012	0.008	0.020	0.017
C24:1	Nervonic acid	0.029	0.018	0.014	0.031	0.026
Monoenes		40.33	38.37	37.81	39.52	39.39
C18:2, trans	Linoleic acid	0.017	0.008	0.003	0.018	0.017
C18:2, cis		0.679	0.368	0.265	0.605	0.541
C22:2	Brassic acid	0.041	0.022	0.015	0.037	0.032
C18:3n-3	Linolenic acid	0.077	0.040	0.026	0.066	0.059
C20:3n-6	Dihomo-gamma-linolenic acid	0.011	0.006	0.004	0.010	0.009
C20:3n-3	Eicosatrienoic acid	0.007	0.004	0.002	0.007	0.006
C20:4n-6	Arachidonic acid	0.052	0.036	0.028	0.053	0.046
C20:5n-3	EPA	0.248	0.144	0.104	0.232	0.203
C22:6n-3	DHA	0.580	0.399	0.298	0.588	0.515
Polyenes		29.12	31.32	31.58	30.48	30.32
Polyunsaturated Fatty Acids		0.88	0.579	0.43	0.873	0.764
